# Supplementary material for: From sequence to enzyme mechanism using multi-label machine learning
Source: BMC Bioinformatics. 2014 May 19;15:150. doi: 10.1186/1471-2105-15-150 (PMC4229970; doi:10.1186/1471-2105-15-150)
Supplement: Additional file 2 — Java code of ml2db. Additional file ml2db_code.tar.gz contains the Java source code to run the multi-label machine learning experiments and save the results to database. The code’s Javadoc is included. [file 1471-2105-15-150-S2.zip › additional file 2/ml2db/ml2db/doc/mulan/classifier/neural/model/Neuron.html]

Neuron


JavaScript is disabled on your browser.


- Overview
- Package
- Class
- Use
- Tree
- Deprecated
- Index
- Help

- Prev Class
- Next Class

- Frames
- No Frames

- All Classes

- Summary:
- Nested |
- Field |
- Constr |
- Method

- Detail:
- Field |
- Constr |
- Method


mulan.classifier.neural.model

## Class Neuron

- java.lang.Object
- - mulan.classifier.neural.model.Neuron

- All Implemented Interfaces:
  :   java.io.Serializable

  ---

    

  ```
  public class Neuron
  extends java.lang.Object
  implements java.io.Serializable
  ```

  Implementation of a neuron unit.
  The neurons are used as processing elements in `NeuralNet`.

  Version:
  :   2012.02.27

  Author:
  :   Jozef Vilcek

  See Also:
  :   Serialized Form

- - ### Constructor Summary

    Constructors

    Constructor and Description

    `Neuron(ActivationFunction function,
    int inputDim,
    double biasValue)`

    Creates a new `Neuron` instance.

    `Neuron(ActivationFunction function,
    int inputDim,
    double biasValue,
    java.util.Collection<Neuron> nextNeurons)`

    Creates a new `Neuron` instance.

    `Neuron(ActivationFunction function,
    int inputDim,
    double biasValue,
    java.util.Random random)`

    Creates a new `Neuron` instance.
  - ### Method Summary

    Methods

    | Modifier and Type | Method and Description |
    | --- | --- |
    | `boolean` | `addAllNeurons(java.util.Collection<Neuron> neurons)` Adds connections to all specified `Neuron` instances. |
    | `boolean` | `addNeuron(Neuron neuron)` Adds a connection to a specified `Neuron`.  The passed instance is assumed to be connected to the output of this instance (forward connections only). |
    | `ActivationFunction` | `getActivationFunction()` Returns the `ActivationFunction` used by the `Neuron`. |
    | `double` | `getBiasInput()` Returns a bias input value. |
    | `double[]` | `getDeltas()` Returns deltas of the `Neuron`. |
    | `double` | `getError()` Returns error term of the `Neuron`. |
    | `double` | `getNeuronInput()` Returns an input value of the `Neuron`. |
    | `double` | `getOutput()` Returns the output of the `Neuron`. |
    | `double[]` | `getWeights()` Returns weights of the `Neuron`. |
    | `double` | `processInput(double[] inputs)` Process an input pattern vector and returns the response of the `Neuron`. |
    | `boolean` | `removeNeuron(Neuron neuron)` Removes a connection to a specified `Neuron`.  The passed instance is assumed to be connected to the output of this instance (forward connections only). |
    | `void` | `reset()` Performs reset, re-initialization of the `Neuron`. |
    | `void` | `setError(double error)` Sets the error term of the `Neuron`. |

    - ### Methods inherited from class java.lang.Object

      `equals, getClass, hashCode, notify, notifyAll, toString, wait, wait, wait`

- - ### Constructor Detail


    - #### Neuron

      ```
      public Neuron(ActivationFunction function,
            int inputDim,
            double biasValue)
      ```

      Creates a new `Neuron` instance.

      Parameters:
      :   `function` - the activation function of the neuron
      :   `inputDim` - the dimension of input pattern vector the neuron can process (the bias not included).
          The input dimension must be greater than zero.
      :   `biasValue` - the bias input value


    - #### Neuron

      ```
      public Neuron(ActivationFunction function,
            int inputDim,
            double biasValue,
            java.util.Random random)
      ```

      Creates a new `Neuron` instance.

      Parameters:
      :   `function` - the activation function of the neuron
      :   `inputDim` - the dimension of input pattern vector the neuron can process (the bias not included).
          The input dimension must be greater than zero.
      :   `biasValue` - the bias input value
      :   `random` - the pseudo-random generator to be used for computations involving randomness.
          This parameter can be null. In this case, new random instance with default seed will be constructed where needed.


    - #### Neuron

      ```
      public Neuron(ActivationFunction function,
            int inputDim,
            double biasValue,
            java.util.Collection<Neuron> nextNeurons)
      ```

      Creates a new `Neuron` instance.

      Parameters:
      :   `function` - the activation function of the neuron
      :   `inputDim` - the dimension of input pattern vector the neuron can process (the bias not included)
          The input dimension must be greater than zero.
      :   `biasValue` - the bias input value
      :   `nextNeurons` - collection of neurons for which this neuron will be an input.
  - ### Method Detail


    - #### getActivationFunction

      ```
      public ActivationFunction getActivationFunction()
      ```

      Returns the `ActivationFunction` used by the `Neuron`.

      Returns:
      :   the activation function


    - #### getWeights

      ```
      public double[] getWeights()
      ```

      Returns weights of the `Neuron`.   
      The index of returned array corresponds to input pattern dimension + 1 for a bias.
      Weight for a bias is at the end of returned array.

      Returns:
      :   weights of the `Neuron`


    - #### getError

      ```
      public double getError()
      ```

      Returns error term of the `Neuron`.

      Returns:
      :   error term


    - #### setError

      ```
      public void setError(double error)
      ```

      Sets the error term of the `Neuron`.

      Parameters:
      :   `error` - the error value


    - #### getDeltas

      ```
      public double[] getDeltas()
      ```

      Returns deltas of the `Neuron`. Deltas are terms, which are used
      to update weights. Here are returned deltas which were computed and used
      to update weights in previous learning iteration.  
      The index of returned array corresponds to input pattern dimension + 1 for a bias.
      Delta for the bias is at the end of returned array.

      Returns:
      :   delta values


    - #### processInput

      ```
      public double processInput(double[] inputs)
      ```

      Process an input pattern vector and returns the response of the `Neuron`.

      Parameters:
      :   `inputs` - input pattern vector

      Returns:
      :   the output of the `Neuron`


    - #### getOutput

      ```
      public double getOutput()
      ```

      Returns the output of the `Neuron`.
      The output value is cached from processing of last input.

      Returns:
      :   output of the `Neuron` or 0 if no
          pattern was processed yet or neuron is after reset.


    - #### getNeuronInput

      ```
      public double getNeuronInput()
      ```

      Returns an input value of the `Neuron`.
      The value is input pattern multiplied with weights and summed
      across all weights of particular neuron. The output of the
      neuron is then input transformed by activation function.
        
      The input values are cached from last processed input pattern.

      Returns:
      :   the input value of the `Neuron` or 0 if no
          pattern was processed yet or neuron is after reset.


    - #### getBiasInput

      ```
      public double getBiasInput()
      ```

      Returns a bias input value.

      Returns:
      :   the input bias


    - #### addNeuron

      ```
      public boolean addNeuron(Neuron neuron)
      ```

      Adds a connection to a specified `Neuron`.  
      The passed instance is assumed to be connected to the
      output of this instance (forward connections only).

      Parameters:
      :   `neuron` - the neuron which is connected to the output of this instance.

      Returns:
      :   true if specified neuron was successfully connected;
          false if connection already exists

      Throws:
      :   `java.lang.IllegalArgumentException` - in neuron is null


    - #### addAllNeurons

      ```
      public boolean addAllNeurons(java.util.Collection<Neuron> neurons)
      ```

      Adds connections to all specified `Neuron` instances.  
      Each instance of the collection is assumed to be connected to the
      output of this instance (forward connections only).

      Parameters:
      :   `neurons` - the collection of neurons which have to be connected to the output of this instance.

      Returns:
      :   true if at least one of specified neurons was successfully connected;
          false if no connection was made. This means that all instances are already connected.

      Throws:
      :   `java.lang.IllegalArgumentException` - if neurons collection is null


    - #### removeNeuron

      ```
      public boolean removeNeuron(Neuron neuron)
      ```

      Removes a connection to a specified `Neuron`.  
      The passed instance is assumed to be connected to the
      output of this instance (forward connections only).

      Parameters:
      :   `neuron` - the neuron which is connected to the output of this instance.

      Returns:
      :   true if connection to specified neuron was successfully removed;
          false if connection did not exist

      Throws:
      :   `java.lang.IllegalArgumentException` - if neuron is null


    - #### reset

      ```
      public void reset()
      ```

      Performs reset, re-initialization of the `Neuron`.
      The weights are randomly initialized, all state variables
      (error term, neuron output, neuron input and deltas) are discarded.


- Overview
- Package
- Class
- Use
- Tree
- Deprecated
- Index
- Help

- Prev Class
- Next Class

- Frames
- No Frames

- All Classes

- Summary:
- Nested |
- Field |
- Constr |
- Method

- Detail:
- Field |
- Constr |
- Method
